# Magnificamide, a β-Defensin-Like Peptide from the Mucus of the Sea Anemone *Heteractis magnifica*, Is a Strong Inhibitor of Mammalian α-Amylases

**DOI:** 10.3390/md17100542

**Published:** 2019-09-21

**Authors:** Oksana Sintsova, Irina Gladkikh, Aleksandr Kalinovskii, Elena Zelepuga, Margarita Monastyrnaya, Natalia Kim, Lyudmila Shevchenko, Steve Peigneur, Jan Tytgat, Emma Kozlovskaya, Elena Leychenko

**Affiliations:** 1 G.B. Elyakov Pacific Institute of Bioorganic Chemistry, Far Eastern Branch, Russian Academy of Sciences, 159, Pr. 100 let Vladivostoku, Vladivostok 690022, Russia; irinagladkikh@gmail.com (I.G.); alekck96@mail.ru (A.K.); zel@piboc.dvo.ru (E.Z.); rita1950@mail.ru (M.M.); natalya_kim@mail.ru (N.K.); lshev@piboc.dvo.ru (L.S.); kozempa@mail.ru (E.K.); 2 School of Natural Sciences, Far Eastern Federal University, 8, Sukhanova St, Vladivostok 690090, Russia; 3 Toxicology and Pharmacology, University of Leuven (KU Leuven), Campus Gasthuisberg, O&N2, Herestraat 49, P.O. Box 922, Leuven B-3000, Belgium; steve.peigneur@kuleuven.be (S.P.); jan.tytgat@kuleuven.be (J.T.)

**Keywords:** Cnidaria, sea anemones, venom, amylase inhibitors, defensin, diabetes

## Abstract

Sea anemones’ venom is rich in peptides acting on different biological targets, mainly on cytoplasmic membranes and ion channels. These animals are also a source of pancreatic α-amylase inhibitors, which have the ability to control the glucose level in the blood and can be used for the treatment of prediabetes and type 2 diabetes mellitus. Recently we have isolated and characterized magnificamide (44 aa, 4770 Da), the major α-amylase inhibitor of the sea anemone *Heteractis magnifica* mucus, which shares 84% sequence identity with helianthamide from *Stichodactyla helianthus*. Herein, we report some features in the action of a recombinant analog of magnificamide. The recombinant peptide inhibits porcine pancreatic and human saliva α-amylases with Ki’s equal to 0.17 ± 0.06 nM and 7.7 ± 1.5 nM, respectively, and does not show antimicrobial or channel modulating activities. We have concluded that the main function of magnificamide is the inhibition of α-amylases; therefore, its functionally active recombinant analog is a promising agent for further studies as a potential drug candidate for the treatment of the type 2 diabetes mellitus.

## 1. Introduction

Type 2 diabetes mellitus is a widespread disease (~8% of adults), often resulting from a metabolic disorder caused by over-feeding, an unhealthy diet, and physical inactivity [1,2]. It covers all age groups of the population, and recently it has spread epidemiologically among children and adolescents [3,4]. The blood glucose levels of diabetic patients reaches abnormally high values, which leads to serious damage to many body systems, especially nerves and blood vessels, causing heart and kidney diseases, blindness, and even the amputation of limbs [5]. The preferred way to maintain good health for people with type 2 diabetes or prediabetes is a control of the input of glucose from the digestive tract into the blood stream [6,7,8]. For this purpose, the medicines based on inhibitors of pancreatic α-amylase are used. Glucobay^TM^, the active ingredient of which, acarbose, inhibits porcine pancreatic α-amylase (PPA) and human saliva α-amylases (HSA) with Ki’s of 0.797 and 1.265 µM, respectively, is one of the most common of that type of drug [9]. In some countries, medicinal plants are traditionally used to treat diabetes; the study of their composition revealed the presence of low molecular weight substances inhibiting mammalian α-amylases [10,11]. Since the effectiveness of currently existing drugs is limited and they have some side effects, the search for new highly effective inhibitors of pancreatic α-amylase is an attractive goal in the field of drug discovery. 

The medicines based on proteins and peptides are poorly represented in the pharmacological market, but attract the interest of specialists due to their high selectivity and effectiveness, combined with relative safety and good tolerability. A large number of proteinaceous α-amylase inhibitors have been isolated from plants, but they are highly specific and interacted with plant α-amylases to control the breakdown of stored starch or with insect α-amylases for defense [12]. Several very effective proteinaceous inhibitors of mammalian, but not plant or microbial α-amylases were found in bacteria belonging to the genus *Streptomyces* [13,14,15,16]. However, it was shown that α-amylase inhibitors isolated from bacteria, for example, tendamistat (Ki 9−200 pM), have a high immunogenicity due to their β-sandwich fold and cannot be used in clinical practice [17].

Among animals, amylase inhibitors were found only in sea anemones, ancient sessile predators inhabiting marine environment. Helianthamide (PPA, Ki = 100 pM; human pancreatic α-amylase (HPA), Ki = 10 pM), the first representative of a new group of α-amylase inhibitors belonging to the β-defensins family, was isolated from *Stichodactyla helianthus* in 2016 [18]. This inhibitor is very active, and in contrast to tendamistat, has a more compact structure, which significantly decreases the likelihood of an immune response. Recently, as a result of the proteomic analysis of the sea anemone *H. magnifica* mucus, we have revealed that α-amylase inhibitors are major components, numbering dozens isoforms [19]. Major α-amylase inhibitor, magnificamide, was identified and sequenced (44 aa, 4770 Da) [19]. It shares 84% of sequence identity to helianthamide (44 aa, 4716 Da). The biological relevance of the presence of inhibitors of α-amylases in the mucus of Cnidaria, such as the sea anemone *H. magnifica*, remains largely unexplained. It is hypothesized that inhibition of α-amylase activity intervenes with the metabolism of starch, which forms a major source of nutrition for many organisms. Organisms exposed to α-amylase inhibitors, therefore, suffer from a reduced availability of carbohydrates that serve as an energy resource.

The results presented here are a continuation of an in depth study of magnificamide, more precisely, of a recombinant analog of the peptide with a detailed investigation of its biological activity.

## 2. Results 

### 2.1. Peptide Expression and Purification

**Figure 1:**
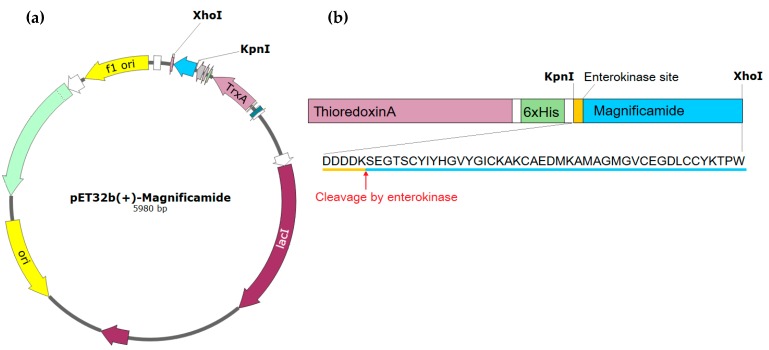
(**a**) Map of the pET32b(+)-magnificamide expression plasmid. A synthetic gene encoding the magnificamide and enterokinase sites was cloned using the restriction sites for KpnI and XhoI. (**b**) The scheme of fusion protein Trx-magnificamide and sequence of magnificamide (UniProtKB—C0HK71).

To study the properties of peptides and then to develop peptide-based drugs, it is necessary to obtain their recombinant analogues at sufficient qualities and quantities. The plasmid vector pET32b(+) containing the gene of thioredoxin ensures high yields of cysteine-containing polypeptides with native conformations, and was, therefore, chosen to create an expression construct. The synthetic gene encoding magnificamide was cloned into pET32b(+) using restriction sites KpnI and XhoI (Figure 1a). The resulting plasmid was transferred into *Escherichia coli* BL21(DE3) cells by electroporation and expressed as a fusion protein Trx-magnificamide (Figure 1b).

**Figure 2:**
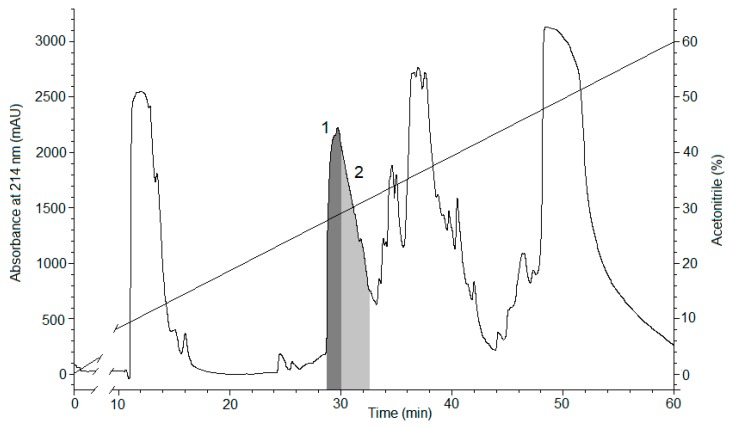
The RP-HPLC elution profile of r-magnificamide, obtained as the result of hydrolysis of the fusion protein Trx-magnificamide by enterokinase, on a Jupiter C4 column (Phenomenex, Torrance, CA, USA) equilibrated by 0.1% TFA, pH 2.2, in a gradient of acetonitrile concentration (0%–70%) for 70 min at 2 mL/min. Fraction 1 containing the mature peptide r-magnificamide (4770 Da) (Figure 3a) is filled by dark grey color; fraction 2 containing peptide with incorrect folding (4777 Da) (Figure 3b) is filled by light grey color.

**Figure 3:**
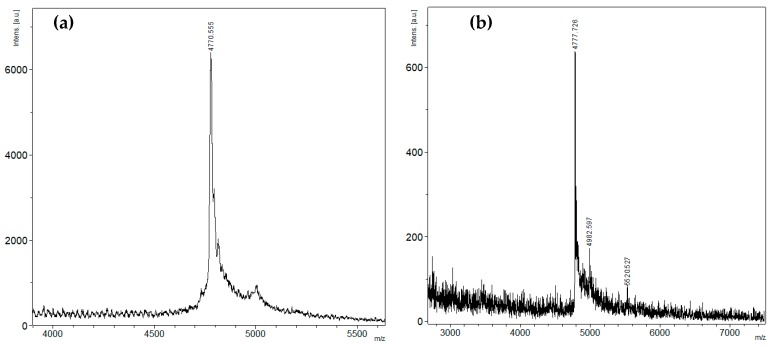
Mass spectra, *m*/*z*, of the peptides isolated by RP-HPLC (Figure 2): (**a**) mature r-magnificamide from fraction 1 and (**b**) incorrectly folded r-magnificamide from fraction 2. *m*/*z*—mass-to-charge ratio; a. u.—arbitrary units.

The fusion protein was isolated from the cell lysate by metal affinity chromatography, desalted, hydrolyzed by enterokinase, and then the recombinant magnificamide (r-magnificamide) was purified by RP-HPLC (Figure 2). After HPLC two fractions which inhibited PPA were obtained, one of them contained the mature r-magnificamide (Figure 3a); the other one contained peptide with incorrect folding (Figure 3b). The average yield of target peptide was equal to 4 mg per 1 L of cell culture (OD A_600_ = 0.6–0.8).

### 2.2. Secondary Structure of Peptides

**Figure 4:**
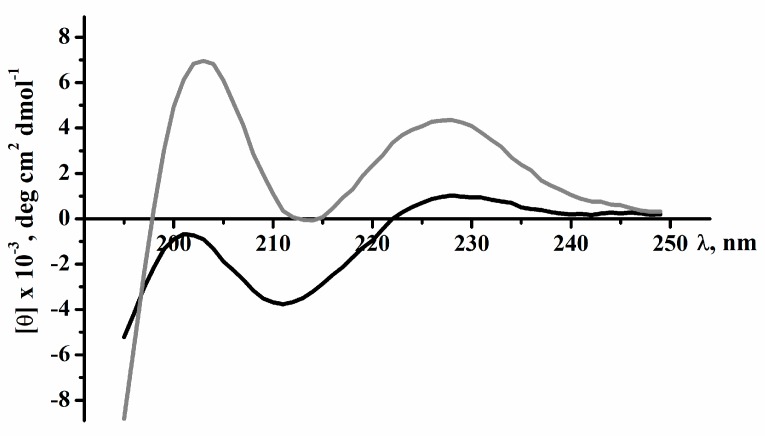
Circular dichroism (CD) spectra of native magnificamide (black line) and recombinant magnificamide (grey line) in 0.01M phosphate buffer, pH 7.0, far or peptide bond UV region.

To calculate the secondary structural elements of recombinant and native magnificamide, the circular dichroism spectroscopy method was used. The spectra in the far UV region (190–240 nm) were characterized by a minimum at 212 nm and a maximum at 203 nm. In the 225–235 nm range, distinct shoulders were observed on the spectra’s curves due to the contribution of the disulfide groups’ absorption (Figure 4). The similarity between the circular dichroism (CD) spectra of magnificamide and its recombinant analogue suggested that the recombinant peptide should be functional and can be used successfully to study its biological activity. Moreover, the calculation of secondary structure elements using Provenzer–Glockner method [20] revealed the complete identities of the peptides at the secondary structure level (Table 1). It should be noted that content of α-helices of the magnificamide was significantly lower than that of helianthamide, which might be reflected in the difference in their spatial structures and biological activity.

### 2.3. Molecular Modeling

**Figure 5:**
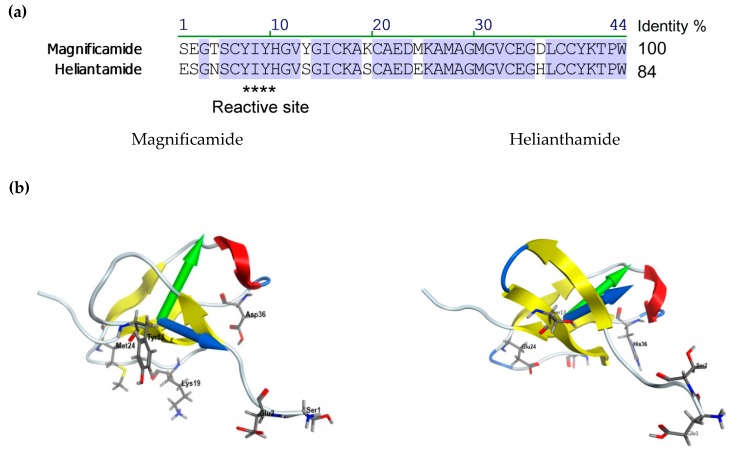

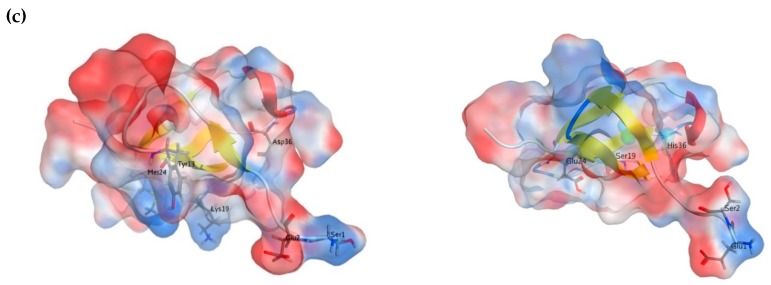
(**a**) Alignment of sea anemone α-amylase inhibitors: magnificamide *H. magnifica* [18] and helianthamide from *S. helianthus* [17] amino acid sequences and their spatial structures. (**b**) The ribbon diagrams of magnificamide and helianthamide spatial structures are colored according to the structure elements; the side chains of the variable residues magnificamide are shown as sticks and labeled. Molecular dipole and hydrophobic moments are indicated by blue and green arrows, respectively. (**c**) Magnificamide and helianthamide molecular surfaces are colored according to surface charge distribution.

The spatial structure models of magnificamide were generated using the homology modeling approach with MOE 2016.08 software (Montreal, QC, Canada) [21]. The atom coordinates of the helianthamide from *S. helianthus*, extracted from the complex with porcine pancreatic α-amylase (PDB ID 4XON) were used as a template. Then the solvated models were optimized using the 400 ns MD simulations in Amber10: EHT force field and the most energetically favorable state of magnificamide was selected (Figure 5b). The molecule has a characteristic fold stabilized by 3 disulfide bridges, including a β-sheet, formed by four strands, an α-helix, and several loops, as well as sufficiently mobile C and N-terminal regions. The content of secondary structure elements agrees well with the data of CD spectroscopy of the native peptide. Despite the relatively high RMSD value for 44 Cα atoms of magnificamide model relative to the prototype—2.46 Å, the model quality assessment showed no conformational constraints (ψ and φ-angles) of amino acid residues, which indicates the good quality of the generated model. It turned out that the largest deviation (from 2 Å to 5.6 Å) involved the flexible parts of the structure in the sequence regions 1–4, 10–12, 18–20, and 32–33. It should be noted that these areas either included variable amino acid residues or were localized in close proximity to those (Figure 5a). The mapping of magnificamide variable residues revealed an interesting feature. In fact, the variability affected only one part of the molecule, while another one remained conservative.

Using the MOE 2016.08 program, the physicochemical characteristics of the inhibitor were evaluated and the surface properties of magnificamide were analyzed to compare them with helianthamide (Table 2). It was shown that, despite its greater compactness, this molecule was characterized by a larger hydrophobic surface area, as well as a redistribution of the localization of charged regions (Figure 5c). This is manifested in a change in both the magnitude and direction of the dipole, and in the hydrophobic moments of the molecules (Figure 5b; Table 2).

### 2.4. Study of Antimicrobial Activity

Since the main function of defensins in most organisms-producers is the protection against microorganisms [22,23,24,25,26,27,28], we performed a screening of potential antimicrobial activity displayed by r-magnificamide. It did not reveal activity against fungi, Gram-positive or Gram-negative bacteria (Table 3).

### 2.5. Study of Channel Modulating Activity

Since defensins are widely present in animal venoms, also known as toxins with modulating effects on the activity of ion channels [23,29,30,31,32,33], we performed an extensive electrophysiological screening of r-magnificamide against 18 subtypes of voltage-gated potassium and voltage-gated sodium channels (mammalian channels: K_v_1.1, K_v_1.2, K_v_1.3, K_v_1.4, K_v_1.5, K_v_1.6, K_v_2.1, K_v_3.1, K_v_4.2, K_v_10.1, hERG, Na_v_1.2, Na_v_1.4, Na_v_1.5, Na_v_1.6 and Na_v_1.8; insect channels: Shaker and BgNa_v_1) (Table 4). r-Magnificamide did not reveal ion channel modulating activity, from which it can be surmised, to conclude, that the main biological function of magnificamide is the inhibition of α-amylases.

### 2.6. Study of Mammalian α-Amylase Inhibition 

Since magnificamide shared a high sequential and structural similarity with the competitive tight-binding inhibitor helianthamide, it was predicted to possess analogous kinetic features. For a quantitative assessment of tight-binding inhibitor potency, Morrison’s method was applied [34,35]. This method uses the Morrison quadratic equation (Equation (1)) for fitting inhibitor-response data and determining Ki *app* as a nonlinear regression parameter.

(1) $$\frac{\upsilon }{\upsilon_{o}}=1-\frac{\left(\left[E\right]+\left[I\right]+K_{i}^{app}\right)-\sqrt{{\left(\left[E\right]+\left[I\right]+K_{i}^{app}\right)}^{2}-4\left[E\right]\left[I\right]}}{2{\left[E\right]}_{o}}$$

True Ki values are calculated from the Equation (2), suggesting a competitive mode of inhibition.

(2) $$K_{i}^{app}=K_{i}\left(1+\frac{\left[S\right]}{K_{M}}\right)$$

**Figure 6:**
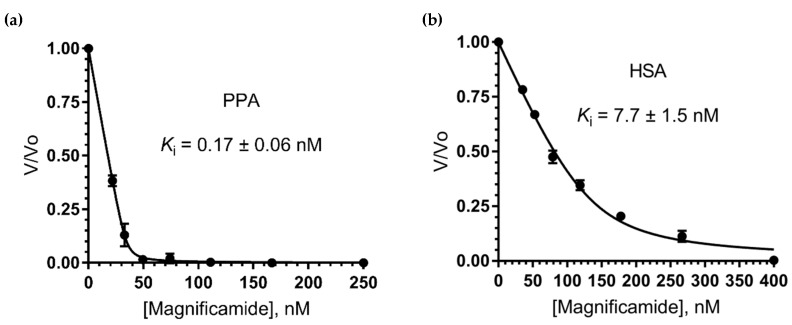
Amylase inhibition curves using r-magnificamide. Fixed concentrations of each enzyme (PPA on (**a**) and HSA on (**b**)) were mixed with increasing concentrations of r-magnificamide (displayed in nM). Each connecting line represents the best fits to the quadratic Morrison equation for tight binding inhibitors [35].

Recombinant magnificamide inhibition constants against porcine pancreatic α-amylase (PPA) and human salivary α-amylase (HSA) were determined. Kinetic assays revealed that recombinant magnificamide was indeed a potent nanomolar tight-binding inhibitor: Ki against PPA was 0.17 ± 0.06 nM; Ki against HSA was 7.7 ± 1.5 nM (Figure 6).

## 3. Discussion

Sea anemones are ancient sessile predators inhabiting the marine environment. They have specialized stinging cells which contain venom rich in peptides acting on different biological targets, mainly cytoplasmic membranes [36,37,38] and ion channels [39,40,41,42,43,44]. Venom with such a complex composition ensured the existence of sea anemones for millions of years [45]. Recently, it has been shown that sea anemones also present a source of pancreatic α-amylase inhibitors belonging to the β-defensin family [18,19]. In the venoms of sea anemones, the β-defensin fold is widely recruited to create toxins modulating ion channel activity, interestingly, often with little amino acid sequence identity, but with similar spatial structure. According to Mitchell and coauthors, cnidarian β-defensin-like toxins can be divided into four main groups: APETx-like, BDS-like, Nv1-like, and ShI-like [46]. Representatives of APETx-like and BDS-like groups interact with ASICs, hERG, voltage-gated sodium, and potassium ion channels [33,47,48,49]. Some of them, crassicorin I and II from *Urticina crassicornis*, reveal paralytic activity against crustaceans, as well as antimicrobial activity against Gram-positive and Gram-negative bacterial strains [33]. Nv1-like and ShI-like peptides are often a major content of sea anemones’ venom and modulate voltage-gated sodium ion channels [50,51]. Helianthamide-like peptides represent a separate group [46] suggesting a different activity.

Taking into account the wide variety of sea anemone defensin-functions, we conducted a study of activity of magnificamide on various ion channels (Table 4) and found no activity. No antimicrobial activity against Gram-positive, Gram-negative bacteria, or fungi was observed either (Table 3). Thus, structural remoteness may occur due to narrow specialization of sea anemones’ helianthamide-like peptides. The presence of numerous digestive enzyme (proteinases and amylases) inhibitors [19] in sea anemone venoms is per se an interesting defensive strategy, similar to plant protection from insects and herbivores.

From a practical point of view, pancreatic α-amylase inhibitors effectively control the influx of glucose into the bloodstream from the gastrointestinal tract [18,19]. Inhibitors of pancreatic α-amylase have a great pharmacological potential for the prevention and treatment of metabolic disorders and type 2 *diabetes mellitus*. In this work we have shown that magnificamide was an effective inhibitor of mammalian α-amylases. The homologue of magnificamide, helianthamide from *S. helianthus*, inhibited PPA with very close Ki (Table 5). Sea anemone inhibitors had great inhibitory activity against mammalian α-amylases (Table 5); the combination of such activity with a compact fold could be used to create new drugs.

Moreover, for the first time, sea anemone peptides’ ability to inhibit HSA was clarified on the example of magnificamide, with Ki equal to 7.7 nM. Inhibition of salivary α-amylase allows for blocking the digestion of starch upon the first stages of entering the body. In addition, it may be useful for the treatment of diseases the of oral cavity, including caries. Caries is a multifactorial disease, a significant role in the development of which is played by oral *Streptococci*, capable of binding salivary α-amylase and using sugar that can be broken down by it for their own needs. The binding of *Streptococci* to salivary α-amylase also contributes to the formation of biofilms and the demineralization of teeth [52]. It has been shown that cherry and tea extracts exhibiting inhibitory activity for salivary α-amylase could inhibit the growth of oral *Streptococci* (in particular *Streptococcus mutans*) [53,54,55]. Given a stable structure and high activity of magnificamide, it may also find an application in the form of chewing gum, as was shown for cherry extract [53].

## 4. Materials and Methods 

### 4.1. Obtaining the Recombinant Magnificamide

The synthetic gene encoding the target peptide was cloned into a pET32b(+) (Novagen, Germany) vector using the restriction sites for KpnI and XhoI by JSC "Eurogen" company, Moscow, Russia. The resulted construct was checked by sequencing to verify the open reading frame. 

The expression construction pET32b(+)/*magnificamide* was used for the transformation of BL21(DE3) *E. coli* cells by electroporation using a Multiporator (Eppendorf, Hamburg, Germany) device. Cells were screened on LB agarose plates containing 100 µg/mL carbenicillin (Invitrogen, Carlsbad, CA, USA). Next the transformed cells were cultured in LB medium (1 L) containing 100 µg/mL carbenicillin at 37 °C to the optical density of (at A_600_) 0.6–0.8. Isopropyl-β,D-thiogalactopyranoside (IPTG) (Invitrogen, Carlsbad, CA, USA) was added to a final concentration of 0.2 mM for induction of expression. The cells were grown for 16 h at 18 °C for the production of the fusion protein in a soluble form and then cells were precipitated from the culture medium by centrifugation at 8000 rpm for 6 min. Cells were lysed by ultra-sonication on a Sonopuls 2070 instrument (Bandeling, Berlin, Germany). The fusion protein Trx-magnificamide was isolated from the cell lysate by metal affinity chromatography on the Ni-NTA-agarose (Qiagen, Hilden, Germany) in native conditions. Then the fusion protein was desalted using Amicon Ultra-15 Centrifugal Filter Units 3000 NMWL (Millipore, Burlington, MA, USA), followed by hydrolysis by Enterokinase (NEB, Ipswich, MA, USA) according to the manufacturer’s instructions. Recombinant magnificamide was purified from the reaction mixture by RP-HPLC, using a Jupiter C_4_ column (Phenomenex, Torrance, CA, USA), equilibrated with 0.1% TFA at pH 2.2, in a gradient of acetonitrile (with concentrations from 0%–70%) for 70 min at 2 mL/min. The retention time of the target peptide was 30 min.

### 4.2. Mass Spectrometry Analysis

MALDI-TOF MS spectra of the peptide fractions obtained by RP-HPLC were recorded using an Ultra Flex III MALDI-TOF/TOF mass spectrometer (Bruker, Bremen, Germany) with a nitrogen laser (Smart Beam, 355 nm), reflector, and the potential LIFT™ tandem modes of operation. Sinapinic acid was used as a matrix. An external calibration was employed using a peptide sample [56] with *m*/*z* 6107 Da and its doubly-charged variant at *m*/*z* 3053 Da. 

### 4.3. Assay of Porcine Pancreatic α-Amylase Inhibitory Activity

The porcine α-amylase (PPA) inhibitory activity of the peptide fractions obtained by RP-HPLC was tested by the following procedure. Experimental samples (10 µL) were added to 80 µL of 50 mM sodium phosphate, 100 mM sodium chloride buffer (pH 7.0), and PPA (A4268) (1 µg/mL) (Sigma Aldrich, St. Louis, MO, USA), and incubated for 10 min at RT. The substrate solution, 2-chloro-4-nitrophenyl α-D-maltotrioside (CNPG3) (Sigma Aldrich, St. Louis, MO, USA), was added to the reaction mixture (1 mM) and incubated for 10 min at RT. The optical absorption was measured on an xMark microplate spectrophotometer (BioRad, Hercules, CA, USA) at 405 nm. Acarbose (1mg/mL) (Sigma Aldrich, St. Louis, MO, USA) was used as a positive control.

### 4.4. Circular Dichroism Spectroscopy

Circular dichroism (CD) spectra were recorded on Chirascan-plus CD spectropolarimeter (Applied Photophysics, Leatherhead, UK) in quartz cells with an optical path length of 0.1 cm for the peptide region spectrum. The cuvette with the solution of the native and recombinant peptides (50 µg/mL) in a 0.01 M sodium phosphate buffer was incubated at 25 °C for 20–25 min before recording the CD spectrum. The content of secondary structure elements of peptides was calculated by the Provenzer–Glockner method [20], using advanced Provencher calculation programs from the CDPro software package (Leatherhead, UK) [57].

### 4.5. Homology Modeling

The generation of a theoretical model of the spatial structure of magnificamide, as well as the valuation of its physico-chemical characteristics, were performed using the specialized software MOE 2016.08 (Montreal, QC, Canada) [21]. Simulation of the molecular dynamics of magnificamide in an aqueous environment, as well as the physicochemical characteristics valuation of α-amylase inhibitors, were performed in Amber10: EHT force field. 

### 4.6. In Vitro Antimicrobial Activity Assay 

The antimicrobial activity of r-magnificamide was tested against Gram-positive (*Staphylococcus aureus* ATCC 21027 and *Bacillus subtilis* ATCC6633), Gram-negative bacteria (*Escherichia coli* VKPM B-7935 and *Pseudomonas aeruginosa* ATCC 27853), and the fungus *Candida albicans* KMM 455 by the agar dilution method. Microbial strains were taken from the American Type Culture Collection (ATCC), Russian National Collection of Industrial Microorganisms (VKPM), and Collection of Marine Microorganisms (KMM) (Pacific Institute of Bioorganic Chemistry FEB RAS). To obtain a microbial lawn, 0.1 mL of a cell suspension (0.5 × 10^8^ cells/mL) was uniformly distributed on agar surface in Petri dishes (15 g/L tryptic soy broth, 2 g/L bacto yeast extract, 1 g/L glucose, and 20 g/L agar for bacteria; for fungi 10 g/L of glucose was added). Wells with a diameter of 6 mm were punched into the agar and filled with 100 µL of the peptide solution at concentrations 1, 5, 10, and 20 µM. The plates were then incubated for 18 h at 37 °C for bacteria, and at 30 °C for the fungus. Minimum inhibitory concentration was determined by measuring the clear zone of inhibition around each well. All assays were performed independently three times.

### 4.7. Electrophysiology

For the expression of Na_v_ channels (mammalian rNa_v_1.2, rNa_v_1.4, hNa_v_1.5, mNa_v_1.6 and hNa_v_1.8 channels; the insect channel *Bg*Na_v_1 from *Blattella germanica*; and the auxiliary subunits rβ1, hβ1, and TipE) and Kv channels (mammalian rK_v_1.1, rKv1.2, hKv1.3, rKv1.4, rK_v_1.5, rK_v_1.6, rK_v_2.1, hK_v_3.1, rK_v_4.2, hK_v_10.1, and h*ERG*; and *Drosophila Shaker*’s IR) in *Xenopus laevis* oocytes; the linearized plasmids were transcribed using the T7 or SP6 mMESSAGE-mMACHINE transcription kit (Ambion, Carlsbad, CA, USA). The harvesting of stage V–VI oocytes from anesthetized female *Xenopus laevis* frog was previously described [58]. Oocytes were injected with 50 nL of cRNA at a concentration of 1 ng/nL using a microinjector (Drummond Scientific, Broomall, PA, USA). The oocytes were incubated in a solution containing 96-mM NaCl, 2-mM KCl, 1.8-mM CaCl_2_, 2-mM MgCl_2_, and 5-mM HEPES (pH 7.4), supplemented with 50 mg/L gentamycin sulfate.

Two-electrode voltage-clamp recordings were performed at room temperature (18–22 °C) using a Geneclamp 500 amplifier (Molecular Devices, Downingtown, PA, USA) controlled by a pClamp data acquisition system (Axon Instruments, Union City, CA, USA). Whole cell currents from oocytes were recorded 1–4 days after injection. The bath solution’s composition was 96-mM NaCl, 2-mM KCl, 1.8-mM CaCl_2_, 2-mM MgCl_2_, and 5-mM HEPES (pH 7.4). Toxins were applied directly to the bath. Resistances of both electrodes were kept between 0.8 and 1.5 MΩ. The elicited currents were sampled at 20 kHz (Na_v_) or 2 kHz (K_v_), filtered at 2 kHz (Na_v_) or 0.5 kHz (K_v_) using a four-pole low-pass Bessel filter. Leak subtraction was performed using a -P/4 protocol. Only data obtained from cells exhibiting currents with peak amplitudes below 2 µA were considered for analysis. For the electrophysiological analysis, a number of protocols were applied from a holding potential of −90 mV with a start-to-start interval of 0.2 Hz. K_v_1.1–K_v_1.6 and Shaker currents were evoked by 250-ms depolarizations to 0 mV followed by a 250 ms pulse to −50 mV from a holding potential of −90 mV. Current traces of hERG channels were elicited by applying a +40 mV prepulse for 2 s followed by −120 mV for 2 s. K_v_2.1, K_v_3.1, and K_v_4.2 currents were elicited by 250 ms pulses to +20 mV from a holding potential of −90 mV. K_v_10.1 currents were evoked by 2-s depolarizing pulses to 0 mV from a holding potential of −90 mV. Sodium current traces were evoked by 100-ms depolarization to the voltage corresponding to maximal sodium current in control conditions. All data were analyzed using pClamp Clampfit 10.0 (Molecular Devices, Downingtown, PA, USA) and Origin 7.5 software (Originlab, Northampton, MA, USA).

### 4.8. Determination of Ki Against Porcine Pancreatic and Human Saliva α-Amylases

Kinetic assays were carried out at 37 °C in 50 mM sodium phosphate and 100 mM sodium chloride (pH 7.0). 2-Chloro-4-nitrophenyl-α-maltotrioside (CNPG3) (Sigma Aldrich, St. Louis, MO, USA) was used as the substrate and the optical absorption of the 2-chloro-4-nitrophenol was measured at 405 nm. Reactions were run with final [CNPG3] = 1 mM ([*S*]/*K*_M_ = 1.41, *K_M_* = 0.71 mM), nominal [*E*] = 20 nM for PPA, [CNPG3] = 3.3 mM ([*S*]/*K_M_* = 0.97, *K_M_* = 3.4 mM), and nominal [*E*] = 100 nM for HSA to provide sufficient analytical signal. Inhibitor dilution schemes were optimized considering recommendations in [34].

The enzyme was pre-incubated with the inhibitor for 10 minutes before the addition of substrate which launched the reaction. Reactions were monitored on an xMark microplate spectrophotometer (BioRad, USA) in the kinetic mode for 30 min. The initial linear steady state region provided initial rate values for each inhibitor concentration (*υ*), along with uninhibited rate values (*υ_o_*). Measurements were run in triplicate. Nonlinear least squares regression was carried out with GraphPad Prism 7.00 (San Diego, CA, USA). Fractional rates (*υ/υ_o_*) were plotted against inhibitor concentrations and the set of data points was fitted by the Morrison Ki regression algorithm [34,35]. Ki and [*E*] were simultaneously treated as adjustable parameters following the approach described in [59]. Derived enzyme active sites concentrations showed physically meaningful values close to nominal (34.6 and 132.5 nM for PPA and HSA respectively). Best-fit constant values were presented as mean ± SE (*n* = 3).

## Figures and Tables

**Table 1:** Secondary structural elements of the natural and recombinant magnificamide and helianthamide (percentages).

Sample	α-Helix			β-Structure			β-Turn	Unordered Structure
	I	II	III	I	II	III		
Magnificamide	0.0	1.7	1.7	21.4	13.7	35.1	24.3	38.9
r-Magnificamide	0.1	1.1	1.2	18.2	14.7	32.9	22.2	43.7
Helianthamide			19			32	18	31
r-Helianthamide			11			33	23	33

**Table 2:** Physico-chemical characteristics of the α-amylase inhibitors.

Physico-Chemical Characteristics	Magnificamide	Helianthamide (PDB ID 4XON)
Radius of hydration (Å)	10.25	10.21
Hydrophilic surface area (Å^2^)	2009.0	1976.3
Hydrophobic surface area (Å^2^)	1553.0	1275.4
WDW volume (Å^3^)	3727.1	4157.9
Isoelectric point	5.81	5.33
Charge	−1.21	−1.82
Dipole moment (D)	161.86	96.07
Hydrophobic moment	145.7	165.9

**Table 3:** Antimicrobial activity of r-magnificamide.

Organisms		r-Magnificamide (1, 5, 10, 20 µM)
Gram-positive	*Staphylococcus aureus* ATCC 21027	Not active
	*Bacillus subtilis* ATCC6633	Not active
Gram-negative	*Escherichia coli* VKPM B-7935	Not active
	*Pseudomonas aeruginosa* ATCC 27853	Not active
Fungi	*Candida albicans KMM* 455	Not active

**Table 4:** Electrophysiological study of r-magnificamide.

Channels		r-Magnificamide (10 µM)
Voltage-gated potassium channels	K_v_1.1, K_v_1.2, K_v_1.3, K_v_1.4, K_v_1.5, K_v_1.6, K_v_2.1, K_v_3.1, K_v_4.2, K_v_10.1, hERG, Shaker *	Not active
Voltage-gated sodium channels	Na_v_1.2, Na_v_1.4, Na_v_1.5, Na_v_1.6, Na_v_1.8, BgNa_v_1 *	Not active

* insect channels.

**Table 5:** Mammalian α-amylase inhibitors from different sources.

Inhibitor name	Source	Mr, Da	Ki, M	Enzyme	Reference
Peptides					
Magnificamide	*H. magnifica*	4770	1.7 × 10^−10^ 7.7 × 10^−9^	PPA HSA	
Helianthamide	*S. helianthus*	4716	1 × 10^−10^ 1 × 10^−11^	PPA HPA	[18]
Tendamistat (HOE-467A)	*Streptomyces tendae* 4158	7958	9 × 10^−12^	PPA	[13]
Parvulustat (Z-2685)	*Streptomyces parvullus* FH-1641	8129	2.8 × 10^−11^ +	PPA HSA	[16]
Low molecular compounds					
Acarbose	*Actinomycetes*	646	0.6 × 10^−6^ 1.3 × 10^−6^	PPA HSA	[9]
Montbretin A	*Crocosmia* sp.	789	8.1 × 10^−9^	HPA	[11]
